# Role of Information Sources in Vaccination Uptake: Insights From a Cross-Sectional Household Survey in Sierra Leone, 2019

**DOI:** 10.9745/GHSP-D-21-00237

**Published:** 2022-02-28

**Authors:** Shibani Kulkarni, Paul Sengeh, Victor Eboh, Mohammad B. Jalloh, Lansana Conteh, Tom Sesay, Ngobeh Ibrahim, Pa Ousman Manneh, Reinhard Kaiser, Yuka Jinnai, Aaron S. Wallace, Dimitri Prybylski, Mohamed F. Jalloh

**Affiliations:** a Global Immunization Division, U.S. Centers for Disease Control and Prevention, Atlanta, GA, USA.; b Oak Ridge Institute for Science and Education, Oak Ridge, TN, USA.; c FOCUS 1000, Freetown, Sierra Leone.; d Sierra Leone Ministry of Health and Sanitation, Freetown, Sierra Leone.; e UNICEF, Freetown, Sierra Leone.; f U.S. Centers for Disease Control and Prevention, Freetown, Sierra Leone.

## Abstract

**Introduction:**

There is limited understanding of the potential impact of information sources on vaccination attitudes and behaviors in low-income countries. We examined how exposure to immunization information sources may be associated with vaccination uptake in Sierra Leone.

**Methods:**

In 2019, a household survey was conducted using multistage cluster sampling to randomly select 621 caregivers of children aged 12–23 months in 4 districts in Sierra Leone. We measured exposure to various sources of immunization information and 2 outcomes: (1) vaccination confidence using an aggregate score (from 12 Likert items, informed by previously validated scale) that was dichotomized into a binary variable; (2) uptake of the third dose of diphtheria-pertussis-tetanus-hepatitis B-*Haemophilus influenzae* type-b-pentavalent vaccine (penta-3) based on card record or through caregiver recall when card was unavailable. Associations between information sources and the outcomes were examined using modified Poisson regression with robust variance estimator.

**Results:**

Weighted estimate for penta-3 uptake was 81% (75.2%–85.5%). The likelihood of uptake of penta-3 was significantly greater when caregiver received information from health facilities (adjusted prevalence ratio [aPR]=1.26, 95% confidence interval [CI]=1.1, 1.5), faith leaders (aPR=1.16, 95% CI=1.1, 1.3), and community health workers (aPR=1.13, 95% CI=1.003, 1.3). Exposure to greater number of information sources was associated with high penta-3 uptake (aPR=1.05, 95% CI=1.02, 1.1).

**Discussion:**

Immunization information received during health facility visits and through engagement with religious leaders may enhance vaccination uptake. Assessments to understand context-specific information dynamics should be prioritized in optimizing immunization outcomes.

## Abstract (precis)

Our findings suggest that health workers and faith leaders are important sources of information to deliver vaccination messages, given their strong association with vaccination confidence and uptake. In this context, vaccination promotion efforts that integrate faith leaders and health workers may help increase vaccination uptake.

## Teaser

**Key Findings:**

- Exposure to vaccination information from faith leaders and health facilities was associated with increased likelihood of vaccination uptake.
- The significant association between exposure to a greater number of immunization information sources and increased likelihood of vaccination uptake reinforces the need for multiple sources to provide consistent and accurate immunization information to facilitate positive vaccination behavior.

**Key Implications:**

- Social and behavior change communication interventions may optimize the promotion of immunization services through multiple information sources such as health facilities and community-based assets including faith leaders and lay community health workers.
- Religion and faith play an important role in how people understand health and make health decisions. In Sierra Leone and other similar settings, interventions to improve uptake of immunization services may be enhanced by proactively engaging faith leaders.

## INTRODUCTION

Sociocultural settings and media environment, along with other contextual factors play an important role in shaping immunization information,^1^^,^^2^ which is delivered via diverse sources (e.g., health workers, television, radio, printed materials, social media, and a myriad of web-based sources).^3^ The content of immunization-related information and how it is received may influence vaccination attitudes and behaviors, either promoting or discouraging the uptake of vaccines.^4^ Negative vaccine content may affect vaccine confidence, which entails trust in the vaccine itself (i.e., efficacy, safety, and product formulation), the health systems that deliver vaccinations, and trust in policies related to vaccines.^5^ Social and behavioral drivers such as perceptions of benefits, safety of vaccines, and social norms related to vaccination, and related motivations can play an important role in vaccination confidence.^6^ Recognizing that context-specific information plays a crucial role in vaccine confidence, it was important to understand in a setting who are the key actors that can be leveraged for messaging. Understanding the association between the exposure to varied sources of information and vaccine confidence and uptake may inform the tailoring of effective immunization communication strategies.

Understanding the association between the exposure to varied sources of information and vaccine confidence and uptake may inform the tailoring of effective immunization communication strategies.

Credible sources of vaccination information such as health workers, community health workers (CHWs), and health organizations can provide accurate, high-quality, and targeted information to caregivers, which can enable them to make informed decisions on vaccination.^7^^,^^8^ On the other hand, rampant misinformation on vaccines, including from sources perceived to be trusted even if not scientifically credible, may discourage or delay vaccination uptake.^9^^,^^10^ Combating misinformation requires the leveraging of credible information sources to create an enabling information environment. For instance, prior studies have shown that caregivers who were exposed to vaccination information from health workers were less likely to express concerns with immunization compared to caregivers who were exposed to information from family, friends, or unscientific literature.^11^^,^^12^

While evidence on the effects of information sources on vaccination uptake exists in high-income countries,^13^ data from the low- and middle-income countries (LMICs) are scarce. However, with the coronavirus disease (COVID-19) vaccination, there is more information now available on information sources and vaccine acceptance.^14^ In the context of the COVID-19 pandemic, new platforms are being deployed to track acceptance of COVID-19 vaccination and identify the potential influential role of information sources. The limited data available on routine immunization and information sources from LMICs are largely consistent with data from the high-income countries. For example, in Nigeria, mothers’ exposure to general child health information via media and community sources increased the likelihood of their children receiving the Bacille Calmette-Guerin (BCG) vaccine.^15^ Similarly, an analysis of Demographic and Health Survey (DHS) data pooled from 13 sub-Saharan African countries showed that mass media use (radio and television) was positively associated with being vaccinated against tuberculosis, polio, diphtheria, tetanus, and pertussis, and measles,^16^ which suggests that information sources may influence vaccination behaviors.

The evidence on exposure to vaccination information and vaccination uptake in LMICs thus far has been limited to general sources of information mainly via DHS data, and prior studies have not specifically measured exposure to immunization-related information.^12^^,^^16^ Contextual factors, such as local beliefs and religious issues, health service delivery system issues, and infrastructure, can also affect availability and exposure to different sources of immunization information. For example, health workers are generally regarded as a reliable source of information in many LMICs, but other structural health systems problems can create barriers to receiving optimal information.^17^ With a complex media and information environment that varies across specific country contexts, it is important to examine how exposure to information may affect vaccination attitudes and uptake to expand our understanding of communication as a social determinant of child vaccination. Mistrust in the vaccines and low knowledge of their health benefits have been identified as critical barriers that resulted in recent outbreaks of vaccine-preventable diseases in LMICs.^18^^,^^19^ Effective communication is one of the core strategies to address this issue.^20^ Increased accurate knowledge about vaccines can improve vaccination uptake behaviors^21^; therefore, understanding the correct strategies and trusted sources for effective vaccine-related communication is crucial.

The evidence on exposure to vaccination information and vaccination uptake in LMICs thus far has been limited to general sources of information mainly via DHS data.

The Government of Sierra Leone has a strong emphasis on community engagement for immunization services. CHWs have traditionally been an integral part of supporting childhood immunization through information sharing, provision for reminders for immunization appointments, and defaulter tracing. Civil society organizations have also played a key role in activities to promote immunization through integration with other maternal and child health activities.^22^^,^^23^ In addition, religious leaders have been instrumental in delivering faith-based immunization messages based on supportive religious text for immunization promotion.^24^

Within this context in Sierra Leone, caregivers of vaccine-eligible children obtain vaccination messages through various sources such as health facilities, organized community events, print materials, radio, television, and social media, and messengers, CHWs, and faith leaders.^25^ Such vaccine promotion strategies have been receiving increased funding and attention from funders and governments over the years. However, there is limited empirical evidence of their associations with vaccination confidence and vaccination uptake.

A key priority for the Sierra Leone Ministry of Health and Sanitation for this assessment was to understand the differential impact of vaccination information sources to prioritize future investments and strategies. The overall objective of this analysis was to understand whether immunization information exposure through different sources may be associated with vaccination confidence among caregivers and affect vaccination uptake among their children. Specifically, we examined the relationship between exposure to (1) type of information source and vaccination confidence, (2) type of information source and vaccination uptake, (3) number of information sources and vaccination confidence, and (4) number of information sources and vaccination uptake.

The Sierra Leone Ministry of Health and Sanitation’s key priority for this assessment was to understand the differential impact of vaccination information sources to prioritize future investments and strategies.

## METHODS

A cross-sectional household survey was conducted in February 2019 in 4 mostly rural districts in Sierra Leone (Kambia, Kono, Moyamba, and Western Area Rural). These districts were selected because they had the lowest coverage of the third dose of pentavalent vaccine-containing diphtheria-pertussis-tetanus-hepatitis B-*Haemophilus influenzae* type-b-vaccine (penta-3) in their respective geographic regions (Kambia district in northern region, Kono district in eastern region, Moyamba district in southern region, and Western Area Rural district in western region).^26^ Penta-3 vaccine coverage in these districts ranged from 69% to 85% based on coverage survey data.^27^

### Sampling and Data Collection

We used a 3-stage sampling design within the 4 districts. In the first stage, clusters (n=72) within each district were selected, using simple random sampling proportional to size across 4 districts. The sample size was determined for the 4 clusters combined (i.e., 1 strata), with an expected coverage of 70% for the penta-3 vaccine. The desired precision was 0.065 with an intraclass correlation coefficient of 0.167, and a response rate of 90%, based on which the calculated sample size was 717. The 2015 census list of enumeration areas served as the sampling frame for the selection of clusters.^28^ In the second stage, all eligible households in each cluster were listed by the enumerators, and 10 households were selected by simple random sampling. If 10 or fewer households were enumerated, all eligible households were included. In the third stage, caregivers of eligible children aged 12–23 months were selected. If 2 or more age-eligible children were present in a household, only 1 of them was selected for inclusion, where enumerators used a random number generator mobile application to select 1 child. Only children age 12–23 months whose date of birth could be confirmed by the caregiver were included in the final sample. We used the World Health Organization (WHO) guidance for sample size calculation for vaccine coverage surveys to determine the sample size.^29^ Data collection was performed by trained interviewers who were from the respective districts and spoke the local language, Krio, fluently. All interviews were conducted in the local language. Interviewers were trained on standardized oral translations of the English questionnaire.

### Outcome Variables

The 2 outcomes examined were: (1) vaccination confidence and (2) uptake of the penta-3 vaccine.

Vaccination confidence was measured using a 12-item questionnaire, which was based on previous validated scale in the African setting and prior literature on vaccine confidence.^30^^,^^31^ The 12-item questionnaire had 10 items on vaccine confidence and 2 items on congruence with religion—each item measured using a 4-point Likert scale with response options ranging from “not at all” (scored 1) to “very much” (scored 4) with 1 exception where the item was used using 3-point response option (Box). Therefore, the total possible score for the items ranged from 18 to 47. To facilitate easier interpretation of the vaccination confidence outcome in subsequent regression analysis, the score was converted to a binary variable by dichotomizing at the unweighted mean to indicate “high vaccination confidence” (above the mean) and “low vaccination confidence” (at or below the mean).

BOX **Questionnaire Used in Sierra Leone on Vaccination Confidence**

Response options for items 1-11: 1. Not at all; 2. Very little; 3. Somewhat; 4. Very much

1. How much do you think vaccines are safe for your child?
2. How much do you think vaccines are good for your child?
3. How much do you think that vaccines protect your child against diseases?
4. How much do you feel confident in your ability to take child for vaccination?
5. How much do people in your community value vaccination services?
6. How much do other parents in your community approve of vaccination?
7. How much does your spouse or partner approves of vaccination?
8. How much of a health threat is measles for children who are unvaccinated?
9. How much do you think illnesses that vaccines prevent are severe?
10. How much does your religion influences vaccination decision for your child?
11. How much would you say childhood vaccination goes together with religious beliefs? Response options for item 12 only: 1. Negatively; 2. Mixed; 3. Positively
12. How do people in your community usually talk about vaccination?

Penta-3 vaccine uptake was measured using a binary variable to indicate whether a child received penta-3 vaccination (coded “1” if received and “0” if not received). The penta-3 vaccine is usually received at 4 months of age. Information on the child’s penta-3 vaccine status was determined at the time of the survey and verified from the child health card given to the caregiver or given through verbal recall of their child’s immunization history. We adapted the standard set of questions used in DHS for obtaining vaccination information through the recall method.^32^

### Explanatory Variables

The main explanatory variable was exposure to information sources. Participants were asked about receiving vaccination information in the past year from 8 potential sources based on settings (community health events, health facilities); channels (radio, TV, social media); and messengers (CHWs, faith leaders). These sources were informed by prior research related to health information in Sierra Leone.^25^ We created a composite count^33^^,^^34^ variable wherein exposure to an information source contributed 1 point; for a total of 8 maximum points indicating exposure to all sources.

Sociodemographic variables related to the caregiver and the child were also included in the assessment. At the individual level, child’s age (months), and mother’s and father’s education variables (binary—none or primary vs. secondary education) were considered because educational status has been shown to be a strong predictor of child health outcomes.^35^^,^^36^ Household size was included because it is known to affect child health (i.e., health-seeking behaviors and resource allocation for child health).^37^ Lastly, whether the child was delivered in a health facility was used as an indicator of health-seeking behavior.

### Statistical Analysis

All analyses were conducted in Stata version 16 (StataCorp LP). Unweighted descriptive statistics (means and proportions) were used to describe the sample sociodemographic characteristics. We used unweighted and weighted statistics to describe exposure to type and number of information sources, vaccination confidence, and penta-3 vaccine uptake. Weighted statistics were obtained using complex survey design accounting for clustering and weighting at household and child levels. We used modified Poisson regression models with robust variance estimation using generalized estimating equations to account for the geographic clustering of caregivers and children within 72 enumeration areas in the 4 districts combined to obtain crude and adjusted prevalence ratio for the cross-sectional data.^38^^,^^39^ We fitted 4 models to examine (1) the association between the type of information source and vaccination confidence, (2) the association between type of information source and penta-3 uptake, (3) the association between the exposure to number of information sources and vaccination confidence, and (4) the association of exposure to the number of information sources and penta-3 vaccine uptake. For the models with type of information source as the explanatory variable, each crude model included whether the caregiver was exposed to a specific type of information source. For adjusted models assessing type of information source, all information sources were added in 1 model to control for exposure to other information sources. All 4 models were adjusted for sociodemographic characteristics of the child’s age, mother’s and father’s education, household size, and facility-based birth. All statistical testing was 2-sided and a *P*-value of <.05 was considered statistically significant. To understand if there was any difference in vaccination uptake outcomes between those with cards and those who provide immunization information based on recall, full models were analyzed for those only based on penta-3 vaccine information on the child health card (Supplement Tables 1 and 2).

### Ethical Approval

Ethical approval for this study was obtained from the Sierra Leone Ethics and Scientific Review Committee. The Human Subjects Office of the U.S. Centers for Disease Control and Prevention approved the assessment as a routine public health activity. All participants gave their verbal informed consent.

## RESULTS

The final sample included in our analysis comprised 621 caregiver-child. The mean child age was approximately 17 months. Half of the mothers (51%) and 46% of the fathers did not have any education. Most children (81%) were delivered in a health facility (Table 1). Immunization history through child health cards was obtained from 588 respondents while 131 self-reported this information. Overall, weighted estimates indicated 78% of caregivers expressed high vaccination confidence and 81% of the children received the penta-3 vaccine.

**TABLE 1.** Sociodemographic Characteristics of the Caregiver-Child Pairs Surveyed in 4 Districts, Sierra Leone, 2019

**Sociodemographic Characteristic**	**No.**	**Percentage **
Household size, mean (SD)	621	7.5 (3.8)
Caregiver characteristics	621	
Mother’s education		
No education	314	50.6%
Primary education	76	12.2%
Secondary education and above	231	37.2%
Father’s education		
No education	277	46.2%
Primary education	31	5.2%
Secondary education and above	292	48.6%
Child characteristics		
Age, mean (SD), months	621	17.3 (3.5)
Birth site	621	
Home or traditional birth attendant	120	19.3%
Health facility	501	80.7%

Abbreviation: SD, standard deviation.

### Information Exposure

Caregivers were on average exposed to approximately 4 information sources. By type of setting, there was greater exposure to health facility visits (84%; 95% confidence interval [CI]=77%, 88%) as compared to community events (33%; 26%–40%). When comparing community-based messengers, exposure to information from CHWs (67%; 59%–75%) was more frequently reported than exposure to information from faith leaders (58%; 48%–68%). Across communication channels, caregivers more frequently received information through the radio (69%; 61%–76%) compared to printed materials (49%; 39%–59%), television (11%; 6%–19%), and social media (10%; 2%–33%). (Table 2).

**TABLE 2.** Unweighted and Weighted Descriptive Statistics for Key Explanatory Variables, Vaccination Confidence, and Uptake of the Penta-3 Vaccine in 4 Districts, Sierra Leone, 2019

**Variables**	No.	**Unweighted Percentage**	**Weighted Percentage (95% CI)** ^a^
Penta-3^b^ uptake	609	77.3%	80.9% (75.2, 85.5)
Vaccination confidence score, mean (SD)	546	43.0 (5.6)	44.4%^c^ (43.7, 45.2)
Vaccination confidence			
High (above mean confidence score)	365	66.5%	77.7% (70.6, 83.6)
Low (at or below mean confidence score)	181	33.2%	22.3% (16.4, 29.4)
Number of information sources, mean (SD)	617	3.62 (1.6)	3.8% (3.4, 4.2)
Exposure to type information sources			
Settings			
Health facility visit	621	85.2%	83.8% (77.3, 88.6)
Community events	621	43.8%	33.0% (26.4, 40.4)
Messenger			
Community health workers	621	71.3%	67.2% (58.7, 74.7)
Faith leaders	621	47.3%	58.1% (47.6, 68.0)
Channels			
Radio	620	66.3%	68.8% (60.8, 75.9)
Printed materials	619	45.6%	48.5% (38.7, 58.5)
Television	620	6.8%	10.6% (5.8, 18.7)
Social media	621	3.5%	10.4% (2.7, 32.5)

Abbreviations: CI, confidence interval; SD, standard deviation.

^a^ Weighted statistics based on complex survey design accounting for clustering, weighting at household and child levels.

^b^ Penta-3 uptake refers to whether a child having received third dose of diphtheria-pertussis-tetanus-Hepatitis B-*Haemophilus influenzae* type-b-pentavalent vaccine; uptake was assessed by card evidence for 488 children and through caregiver recall for 121 children.

^c^ Weighted mean.

#### Association Between the Type of Information Source and Vaccination Confidence

Vaccination confidence was more prevalent among those who received immunization information from faith leaders (adjusted prevalence ratio [aPR]: 1.20; 95% CI=1.1, 1.4). On the other hand, vaccination confidence was negatively associated with exposure to information from CHWs (aPR=0.83; 95% CI=0.7, 0.9) (Table 3).

**TABLE 3.** Crude and Adjusted Prevalence Ratio for the Association Between Exposure to Types of Information Sources With Vaccination Confidence and the Uptake of the Penta-3 Vaccine in 4 Districts, Sierra Leone, 2019

	**Vaccination Confidence**				**Penta-3 Uptake**			
	**N=546** ^a^		**N=527**		**N=609** ^b^		**N=586**	
	cPR (SE)	95% CI	aPR^c^ (SE)	95% CI	cPR (SE)	95%CI	aPR^c^ (SE)	95% CI
Messengers								
Faith leader	1.21 (0.1)	1.1, 1.4	1.20 (0.1)	1.1, 1.4	1.18 (0.1)	1.1, 1.3	1.16 (0.1)	1.1, 1.3
Community health worker	0.92 (0.1)	0.8, 1.02	0.83 (0.05)	0.7, 0.9	1.19 (0.1)	1.05, 1.3	1.13 (0.1)	1.003, 1.3
Settings								
Health facility	1.16 (0.1)	0.9, 1.5	1.16 (0.1)	0.9, 1.5	1.24 (0.1)	1.1, 1.4	1.26 (0.1)	1.1, 1.5
Community health event	1.14 (0.1)	1.01, 1.3	1.11 (0.1)	1.0, 1.2	1.13 (0.1)	1.03, 1.2	0.99 (0.1)	0.9, 1.1
Channels								
Radio	1.09 (0.1)	0.9, 1.3	0.99 (0.1)	0.9, 1.2	1.02 (0.1)	0.9, 1.1	0.94 (0.04)	0.9, 1.03
Printed messages	1.11 (0.05)	1.0, 1.2	0.99 (0.1)	0.9, 1.1	1.1 (0.05)	1.01, 1.2	1.01 (0.05)	0.9, 1.1
Social media	1.18 (0.1)	1.04, 1.3	1.03 (0.1)	0.8, 1.3	1.06 (0.1)	0.8, 1.3	0.91 (0.1)	0.7, 1.2
TV	1.05 (0.1)	0.9, 1.2	1.02 (0.1)	0.9, 1.2	1.11 (0.1)	0.9, 1.2	1.13 (0.1)	1.01, 1.3

Abbreviations: aPR, adjusted prevalence ratio; CI, confidence interval; cPR, crude prevalence ratio; SE, standard error; penta-3, third dose of diphtheria-pertussis-tetanus-Hepatitis B-*Haemophilus influenzae* type-b-pentavalent; Ref, reference.

^a^ Sample size for models with radio, TV, and printed materials=545.

^b^ Sample size for models with radio and TV= 608, and printed materials=607.

^c^ Adjusted model includes adjusting for other information sources and sociodemographic variables (household size, mother’s and father’s education, child age, child’s birth at health facility).

#### Association Between Type of Information Source and Penta-3 Vaccine Uptake

Based on adjusted estimates, caregivers who were exposed to information from health facilities were 26% more likely to have their child vaccinated with the penta-3 vaccine (Table 3). Similarly, there was a significant positive relationship between vaccine uptake and exposure to information from faith leaders (16%) and community health worker and television (13%) each. The positive relationship remained consistent for exposure to faith leaders (aPR=1.08; 95% CI=1.01, 1.1) and health facility visits (aPR=1.29; 95% CI=1.1, 1.5) with penta-3 vaccine uptake when considering only the sample of children possessing a child health card (Supplement Table 1).

#### Association Between Exposure to Number of Information Sources and Vaccine Confidence

Exposure to number of information sources was positively associated with 5% greater likelihood of vaccine confidence in the crude model, but this relationship was not significant in the adjusted model. (Table 4). Mother’s and father’s secondary education compared to no education also increased the likelihood of penta-3 vaccine uptake by 13% and 21%, respectively, in crude models; these relationships were not significant in the adjusted models.

**TABLE 4.** Crude and Adjusted Prevalence Ratios for the Associations Between Exposure to Number Information Sources With Vaccination Confidence and Uptake of the Penta-3 Vaccine in 4 Districts, Sierra Leone, 2019

	**Vaccination Confidence**				**Penta-3 Uptake**			
	**N=546** ^a^		**N=527**		**N=609** ^b^		**N=586**	
	cPR (SE)	95% CI	aPR (SE)	95% CI	cPR (SE)	95% CI	aPR (SE)	95% CI
Number of information sources	1.05 (0.02)	1.01, 1.1	1.03 (0.02)	0.9, 1.07	1.06 (0.02)	1.03, 1.1	1.05 (0.02)	1.02, 1.09
Household size	0.99 (0.01)	0.9, 1.01	1.00 (0.01)	0.9, 1.01	1.01 (0.01)	0.9, 1.03	1.02 (0.01)	1.0, 1.03
Mother’s education								
No education	Ref	Ref	Ref	Ref	Ref	Ref	Ref	Ref
Primary	1.05 (0.1)	0.9, 1.2	1.00 (0.1)	0.9, 1.1	1.07 (0.1)	0.9, 1.2	1.04 (0.1)	0.9, 1.2
Secondary and above	1.13 (0.1)	1.01, 1.3	1.03 (0.1)	0.9, 1.2	1.07 (0.05)	0.9, 1.2	1.03 (0.05)	0.9, 1.1
Father’s education								
No education	Ref	Ref	Ref	Ref	Ref	Ref	Ref	Ref
Primary	1.12 (0.1)	0.9, 1.3	1.10 (0.1)	0.9, 1.3	1.09 (0.1)	0.9, 1.3	1.02 (0.1)	0.9, 1.2
Secondary and above	1.21 (0.1)	1.1, 1.4	1.14 (0.1)	0.9, 1.3	1.08 (0.05)	0.9, 1.2	1.04 (0.05)	0.9, 1.2
Child age, months	0.99 (0.1)	0.9, 1.00	0.99 (0.01)	0.9, 1.0	1.02 (0.01)	1.0, 1.03	1.02 (0.01)	1.0, 1.004
Birth site								
Home or TBA site	Ref	Ref	Ref	Ref	Ref	Ref	Ref	Ref
Health facility	1.17 (0.1)	1.03, 1.3	1.11 (0.1)	0.9, 1.3	1.16 (0.06)	1.04, 1.3	1.07 (0.1)	0.9, 1.2

Abbreviations: aPR, adjusted prevalence ratio; CI, confidence interval; cPR, crude prevalence ratio; SE, standard error; penta-3, third dose of diphtheria-pertussis-tetanus-Hepatitis B-*Haemophilus influenzae* type-b-pentavalent; Ref, reference; TBA, traditional birth attendant.

^a^ Sample size for crude model with number of information sources=543 and for the model with father’s education as the independent variable for vaccination confidence=529.

^b^ Sample size for crude model with number of information sources=605 and for the model with father’s education as the independent variable for penta-3 uptake=589.

#### Association Between Exposure to Number of Information Sources and Penta-3 Vaccine Uptake

Exposure to number of information sources was significantly associated with 5% greater likelihood of penta-3 vaccine uptake (adjusted estimates). Child age and household size were also positively associated with penta-3 vaccine uptake (Table 4). Association between exposure to a greater number of information sources and uptake was not significant in the restricted sample to those with child health cards only (aPR=1.02, 95% CI=0.9, 1.04), (Supplement Table 2).

## DISCUSSION

Our household survey in 4 mostly rural districts in Sierra Leone showed that caregivers were exposed to diverse sources of immunization information that varied by settings, channels, and messengers, and exposure to greater information sources were associated with uptake of the penta-3 vaccine. On average, caregivers were exposed to 4 different information sources. There was greater exposure through health facilities compared to community engagement events, which points to the dominant role of health workers as the primary health communicators. Radio and print materials were among common channels of receiving information. More than half of all caregivers were exposed to information from CHWs and faith leaders. Uptake of penta-3 was positively associated with receiving information from health facilities, faith leaders, and CHWs. High vaccination confidence was positively associated with exposure to immunization information from faith leaders but was negatively associated with exposure to information from CHWs. The associations we found may be suggestive of information sources that need to be prioritized as well as those that need to be strengthened to optimize immunization in Sierra Leone.

Vaccine uptake was positively associated with receiving information from health facilities, faith leaders, and CHWs.

The significant association between exposure to a greater number of information sources and greater prevalence of high vaccination uptake suggest the importance of multiple sources to provide consistent immunization information to improve uptake. Multiple information sources may create positive reinforcement of health messages and help generate social norms that could support child immunization.^40^ Regarding the type of information source, the greatest exposure did not necessarily have the strongest association with penta-3 vaccine uptake in our assessment. In fact, some high exposure sources, such as radio and printed materials, were not at all associated with either outcome. In these mostly rural areas in Sierra Leone, perhaps the physical setting in which the message is received (health facility and community events) as well as the community messengers are more influential than the channels (e.g., radio and printed materials) used to communicate immunization information. Future studies should consider experimental designs to assess the differences in effectiveness across information sources based on their settings, channels, and messengers^41^ including content of the messaging, consistency in messaging, and/or any dose-response relationship between number of times a message is received.

The findings from our study underscore the importance of understanding the role that different types of information sources play in promoting immunization. In these mostly rural districts in Sierra Leone, we found that receiving immunization information from a faith leader was the strongest predictor for expressing high vaccination confidence and was also strongly associated with vaccination uptake. We could not directly discern if exposure to messages from religious leaders had such strong associations with these outcomes because they are trusted sources, but religious leaders have traditionally played an important role in influencing public perception. Messages from religious leaders are respected and followed and have been found to resonate with caregivers’ values and beliefs while conveying the importance of vaccines from their specific sociocultural perspective.^42^^,^^43^ In the 1980s in Sierra Leone, religious leaders led a successful social mobilization strategy that delivered messages drawn from religious scriptures to highlight the importance of vaccines, consequently improving child vaccination coverage.^24^ During the Ebola outbreak in Sierra Leone, the role of faith leaders in public health was strongly highlighted once again when they promoted protective behaviors for safe burials.^44^^,^^45^ Therefore, our findings are consistent with the significant role of the faith leaders as powerful influencers of behavior change. On the other hand, religious opposition to vaccination has been documented in many other settings,^46^ which has contributed to refusal and/or delay of vaccination. Faith leaders and other important community leaders should be actively engaged not only in the delivery of messages but also in the development of messages that align and resonate with the community’s beliefs and values in promoting immunization services to safeguard resilient demand and uptake of life-saving vaccines.

Faith leaders and other community leaders should be actively engaged not only in delivering messages but also in developing messages on immunization that align and resonate with the community’s beliefs and values.

Our findings also emphasize the importance of information obtained at health facilities to increase vaccination uptake. Most caregivers in our study population received immunization information from health facilities. Health facilities serve as a source of information and as sites for vaccination services, which may explain the positive relationship with vaccination uptake. There is strong scientific evidence on the importance of access to health facilities for improving vaccination coverage.^47^^–^^49^ The positive relationship between information from health facilities and high uptake of vaccines may also be indicative of greater health-seeking behaviors, utilization of services, and greater availability of services at a health system level, all of which are related to improved vaccine coverage.^50^^,^^51^ The role of health facilities may also suggest the integral role of health workers as important sources of accurate information to caregivers at the facilities. It has been shown that interaction and communication with health workers are notable factors associated with vaccination uptake in LMICs.^52^^,^^53^ Therefore, our findings could also suggest the important role of interpersonal communication between health workers and caregivers, as well as health worker attitudes and knowledge about vaccination as influencers of vaccination uptake.^54^^,^^55^

Although CHW exposure was negatively associated with vaccination confidence, it was at the same time positively associated with uptake of penta-3. This seemingly conflicting finding may be due to reverse causality observed in our cross-sectional results. In Sierra Leone, and sub-Saharan Africa settings, CHWs are used in targeted vaccination campaigns and community engagement efforts to reach children who may have delayed or missed scheduled vaccine doses due to many reasons including the possibility of low vaccination confidence. Therefore, it is possible that in the 4 mostly rural districts sampled in our survey, CHW efforts may have been intensified in communities with low confidence to help improve vaccination uptake. Given the inability to establish temporality between the exposure and the outcome with the current data, reverse causality is possible. Again, this finding reinforces the need for longitudinal designs in understanding the time-varying impact of exposure to specific sources of immunization on vaccination outcomes.

### Limitations

Our study has several limitations. Due to the observational study design, we cannot make any causal inferences regarding the definitive relationship between exposure to information sources and the uptake that we observed in the sample from Sierra Leone. In addition, it is possible that respondents could have conflated the different information sources when reporting their exposure. For example, information received from a religious leader via a radio program might have been reported only as exposure to the religious leaders without capturing radio as the channel of the information delivery. We found very little variability in the information trust variables, with all information sources being highly trusted by caregivers. While this is a limitation in terms of statistical model fitting purposes, this finding demonstrates that not all self-reported trusted sources translate into having a significant association with vaccination confidence and uptake. Another possible explanation of the lack of variability in the information trust variables may have been due to the response format used in measuring information trust (i.e., yes/no response format). Future assessments should consider using Likert-type items to measure trust in vaccination messages received from information sources to increase the chances of having greater variability in the responses. Finally, we assessed the exposure to information sources without evaluating the content of the information communicated, information quality, or how the information was targeted. We also acknowledge that vaccine uptake is a result of multiple, interrelated health systems, community, and individual-level factors, however, since our data was from a caregiver perspective, we did not have information on system-level factors to examine for this analysis. Future research may consider the use of rigorous experimental designs to assess the impact of exposure to disparate vaccination information sources on the uptake of vaccines. Furthermore, qualitative approaches are needed to get a richer understanding of how people receive, engage with, and act upon the vaccine-related information within local settings.

## CONCLUSION

Depending on the country context, community-level interventions that use trusted sources of information to deliver vaccination messages, such as faith leaders, should be prioritized to increase vaccination uptake. This study in Sierra Leone has implications for measuring and addressing vaccination behaviors in similar LMIC settings. Our findings imply that high level of exposure to immunization information from radio programming and printed materials alone may not sufficiently translate into improving vaccination confidence and uptake. Efforts to optimize vaccination outcomes may benefit from strengthening interpersonal communication during health facility visits and maximizing on community engagement through use of trusted messengers. Our assessment can inform future longitudinal studies to evaluate the causal effect of information exposure on immunization outcomes in low resource settings.

## Supplementary Material

21-00237-Kulkarni-Supplement.pdf

## Notes

**Peer Reviewed**

**First published online:** February 22, 2022.
