# Supplementary material for: Role of Information Sources in Vaccination Uptake: Insights From a Cross-Sectional Household Survey in Sierra Leone, 2019
Source: Glob Health Sci Pract. 2022 Feb 28;10(1):e2100237. doi: 10.9745/GHSP-D-21-00237 (PMC8885335; doi:10.9745/GHSP-D-21-00237)
Supplement: 21-00237-Kulkarni-Supplement.pdf [file 21-00237-Kulkarni-Supplement.pdf]

Supplement to: Kulkarni S, Sengeh P, Eboh V, et al. Role of information sources in vaccination uptake: insights from a cross-sectional household survey in Sierra Leone, 2019. *Glob Health Sci Pract*. 2022;10(1):e2100237. https://doi.org/10.9745/GHSP-D-21-000237

Supplement Table 1: Adjusted prevalence ratios for the association between exposure to types of information sources with uptake of the third dose of pentavalent vaccine based on Child Health Card in four districts, Sierra Leone, 2019

**Source of information**		
	**N=469**	
	aPR (SE)	95%CI
**Messengers**		
Faith leader	1.08 (0.03)	1.01-1.1
Community health worker	1.05 (0.06)	0.9-1.2
**Settings**		
Health facility	1.29 (0.1)	1.1-1.5
Community health event	0.98 (0.05)	0.9-1.1
**Channels**		
Radio	0.94 (0.04)	0.9-1.02
Printed Messages	0.92 (0.03)	0.9-0.99
Social media	0.97 (0.1)	0.8-1.1
TV	1.11 (0.05)	1.02-1.2

NOTE: Adjusted model includes adjusting for other information sources and sociodemographic variables (household size, mother's and father's education, child age, child's birth at health facility).

cPR: crude prevalence ratio; aPR: adjusted prevalence ratio; SE: Standard Error; 95%CI: 95% Confidence Interval

Supplement to: Kulkarni S, Sengeh P, Eboh V, et al. Role of information sources in vaccination uptake: insights from a cross-sectional household survey in Sierra Leone, 2019. *Glob Health Sci Pract*. 2022;10(1):e2100237. https://doi.org/10.9745/GHSP-D-21-000237

Supplement Table 2: Adjusted prevalence ratios for the associations between exposure to number information sources with uptake of the third dose of pentavalent vaccine (penta-3) based on Child Health Card in four districts, Sierra Leone, 2019

	**Penta-3 uptake**	
	**N=469**	
	aPR (SE)	95% CI
**Number of information sources**	1.02 (0.01)	0.9-1.04
**Household size**	1.01 (0.04)	1.0-1.02
**Mother's education**		
No education	Ref	-
Primary	1.02 (0.1)	0.9-1.1
Secondary and above		
**Father's education**		
No education	Ref	-
Primary	0.9 (0.1)	08-1.1
Secondary and above	1.04 (0.05)	0.9-1.1
**Child age (mo)**	1.01 (0.05)	0.9-1.02
**Birth site**		
Home or TBA site	Ref	-
Health facility	1.04 (0.05)	0.9-1.15

TBA: Traditional Birth Attendant; cPR: crude prevalence ratio; aPR: adjusted prevalence ratio; SE: Standard Error; 95%CI: 95% Confidence Interval
